# Current Conveyor All-Pass Sections: Brief Review and Novel Solution

**DOI:** 10.1155/2013/429391

**Published:** 2013-11-28

**Authors:** Sudhanshu Maheshwari

**Affiliations:** Department of Electronics Engineering, Zakir Hussain College of Engineering and Technology, AMU, Aligarh 202002, India

## Abstract

This study relates to the review of an important analog electronic function in form of all-pass filter's realization using assorted current conveyor types and their relative performances, which resulted in a novel solution based on a new proposed active element. The study encompasses notable proposals during last the decade or more, and provides a platform for a broader future survey on the topic for enhancing the knowledge penetration amongst the researchers in the specified field. A new active element named EXCCII (Extra-X second generation current conveyor) with buffered output is found in the study along with its use in a new first-order all-pass section, with possible realization using commercially available IC (AD-844) and results.

## 1. Introduction to the Topic

The first-order electronic function which finds a wide range of applications in analog signal processing and signal generation is a network classified under “filters.” It is an all-pass filter, which is also called a phase shifter, for its amplitude preserving feature (former name) and frequency-dependent phase characteristics (the latter name). The two features together make it a very powerful electronic function with extensive applications. These range from a simple phase equalizer or phase shifter to more complex ones like signal generation with quadrature or multiphase outputs. Moreover, higher order filtering functions can be further realized with the help of this simple electronic function.

When realized using high-performance active element like current conveyor, the first-order all-pass filters become a natural building block for modern communication and instrumentation systems. High accuracy, wide, bandwidth and exceptionally high slew rates combined with low voltage and low power implementations of current conveyors makes it an ideal choice for modern applications in analog signal processing. This prompted researchers worldwide to study this function in details. This work is devoted to a review of a repertoire of author's first-order all-pass filters, more often referred to as all-pass sections, which have appeared in the technical literature little over the last decade. This fulfills the motivation of not only compiling the knowledge contribution by the author in this area, but also bringing together many other useful contributions by other researchers as well. In the process, a new active element named Extra-X second generation current conveyor with buffered output (EXCCII) is also discovered and a new all-pass section emerges as a result. Thus, the work is expected to further the knowledge in this area and open up new avenues for further research.

## 2. Review

Current conveyor-based first-order all-pass filters had been researched to a certain extent at the time when authors' first work was communicated in late 1998. It is apparent from the survey carried out in that work, which appeared in literature in 2000 [1]. The work of [1] provided a new impetus to the research in the area. A second generation current conveyor of negative type (CCII−) was used to realize first-order all-pass function, with a floating capacitor and two grounded resistors. It was, for the first time, that the idea of employing translinear conveyor for all-pass function was also given therein [1]. The single translinear conveyor-based circuit with two passive components was minimal at the time and continues to compete with most recent work, as shall follow later. Soon, third generation current conveyor was next used for realizing not only voltage mode function but also current-mode filters [2]. One of the two voltage-mode circuits in [2] had its gain adjustment (≤1) featured by employing four passive elements in all, whereas the other circuit used same component count as the first [1] work. Current-mode circuit with only two passive elements was a first attempt employing third generation current conveyor (CCIII) [2]. Although for sensing the output current, an additional active element was required. The CCIII-based circuits enriched the application of this active element, which had been only rarely used till then, as is also evident from the references cited therein [2]. A dedicated current-mode paper was then reported using CCIII and two passive elements for four new all-pass sections, with similar output current sensing requirement as before [3]. Almost at the same time, translinear-C current mode all-pass sections also appeared, which used translinear conveyor (current controlled conveyors, CCCII) and capacitor [4]. The circuits in [4] were electronically tunable through bias current of CCCII in a wide frequency range. However, independent access to the output current again required additional current follower. Another work followed where three different circuits were proposed with the feature of operability in either voltage or current mode, without changing the circuit configuration [5]. Starting with a CCII− based network, a translinear conveyor-based circuit and then a translinear-C version were given, along with an application in realizing quadrature oscillator. The translinear-C circuit was electronically tunable. The paper demonstrated the use of a CCCII− for realizing a floating tunable resistor for the first time.

All works mentioned above were based on the use of floating capacitor [1–5]. In 2006, grounded capacitor-based first-order all-pass filter using a relatively more complex active element, fully differential current conveyor (FDCCII) were reported [6]. Six distinct circuits providing first-order functions, with both voltage and current outputs, were proposed. First time use of FDCCII, combined with the multifunctional capability of the circuits, made the work a novel contribution [6]. Later, FDCCII was employed for realizing this function with better characteristics, as shall follow in chronological order.

The year 2007 saw many works in the form of both current and voltage modes. Translinear-C all-pass sections in current-mode with accessible outputs at high impedance nodes enjoyed electronic tunability and high frequency operation [7]. Quadrature oscillator applications confirmed the utility of proposed all-pass sections, but the drawback of employing floating capacitor again turned out to be a blessing in disguise like other work [1–5]. It ensured easy high frequency signal transmission from input to output node through ideally shorted capacitor (at high frequencies), bypassing the active element and hence its frequency limitations. Another current-mode work became available with the advantage of employing two grounded components, easy for integration, and an active element in the form of a modified DVCC with a topology compatibility with CCIII as well [8]. This paper [8] introduced the concept of modified DVCC with a current transfer gain from  *X*  to  *Z*  terminal of “2” instead of traditional “unity” value. However, input current insertion at two nodes necessitated additional current follower block for practical purpose [8]. Next, CDBA-based voltage-mode all-pass filters were reported in the same year with the advantage of low output impedance [9]. CCCDBA had already been proposed by the author; hence, the new circuits [9] were shown to be compatible with the new active element and hence electronically tunable. CCCDBA-based circuits employed only two passive components and provided new directions for further study on tunable realizations using the active element.

None of the voltage-mode circuits discussed so far [1–3, 5, 6, 9] exhibited high input impedance and use of only grounded passive components. The work based on two DVCCs with only  *Z*+ stages and three passive grounded components contributed six circuits each enjoying high input impedance as well [10]. High input impedance allowed easy cascading within a voltage mode system, while grounded components ensured fabrication ease. The former feature was demonstrated in quadrature oscillator design therein [10]. Very soon similar work appeared where six distinct circuits with high input impedance were proposed [11]. The matching requirements in [10] were in a ratio of “2”, whereas the same in [11] required a ratio of “1.” The circuits of [11] were more practical by the way of employing a resistive or capacitive termination at the output  *Z*  node, unlike the circuits of [10], where the output appeared at high impedance node (*Z*) with no terminations. The catalogue of 12 circuits [10, 11] provide a new advancement to the knowledge on DVCC-based realizations, which was further enriched by a current-mode paper with one DVCC and two grounded components [12]. The circuits presented therein provided current outputs at desirable high impedance nodes (*Z*). Other first-order functions were also realized in that work. High impedance current output meant easy cascading within current-mode system, as was also demonstrated in compact quadrature oscillator applications proposed therein [12].

The active element, DVCC, was treated as an analog block (linear) and its applications in digital (nonlinear) were not explored. A very novel work, where DVCC was shown to be not only tunable, but also good for some nonlinear applications [13] was next reported. DVCC's X-terminal resistance was shown to be electronically tunable through the bias voltage of DVCC, so as to be used for a new voltage-controlled all-pass section. The pole-frequency tuning was demonstrated through the bias voltage. The circuit [13] used two DVCCs and one grounded capacitor as the only passive component so as to fall in active-C category, with the feature of electronic tuning and low output impedance as well. An interesting and new application in four phase clock generation was also given, where the DVCCs were used as comparators for the first time. The concept was later popularized for complex neural circuits as well. Furthermore the research on tunable DVCC also got an impetus thereafter.

Coming back to the active-RC circuits, a new topology was discovered, which produced six all-pass filters besides several other first-order simple analog blocks. Each all-pass circuit employed one DVCC with  *Z*+ stage only and three passive components [14]. The work extended beyond first-order functions to second-order filters and third-order oscillators. A true voltage mode work on all-pass filters presented one FDCCII and two components-based circuits with high input and low output impedances, and further demonstrated their utility for higher-order filters and oscillators [15]. The most recent update to the technical literature proposes a new active building block named dual-X current conveyor with buffered output and all-pass filters using this active building block. Four new circuits both use two or three passive elements and enjoy high input as well as low output impedance [16]. As compared to [15], the active element is simpler and hence economical. Moreover, high frequency potential of the new circuits makes them superior to other work [1–15].

## 3. A Novel Solution and Comparisons

The preceding section presented a detailed look at notable first-order all-pass filters published during the last decade. Picking the first and the latest reference shows some interesting revelations [1, 16]. The work based on CCII− [1] and the one with buffered output DXCCII [16] shows a common feature of employing a floating capacitor. For comparison sake, out of the four circuits proposed in [16], the one closest to the first work [1] is chosen; it is Filter-4 of [16]. Both these circuits ([1] and Filter-4 of [16]) use three passive components in the form of a capacitor as already mentioned and two grounded resistors. But what these two circuits differ in is the input and output impedance levels, although the sign of the transfer function also differs. The circuit of [1] does not exhibit high input impedance and low output impedance contrary to the latest ones [16]. Now, comparing the circuit's relative complexities, it is to be mentioned that buffered output DXCCII [16] is complex when compared to a CCII−. Thus, keeping in view the relative complexity and features realized in two extreme time separated work [1, 16], an optimum solution is desired, which is missing in the literature ([1–16] and cited therein).

**Figure 1:**
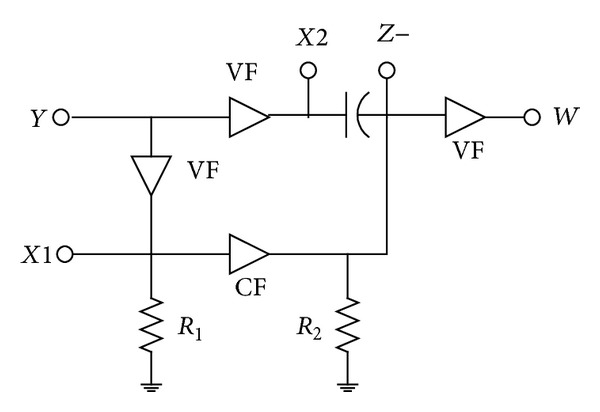
Basic scheme for all-pass section.

**Figure 2:**
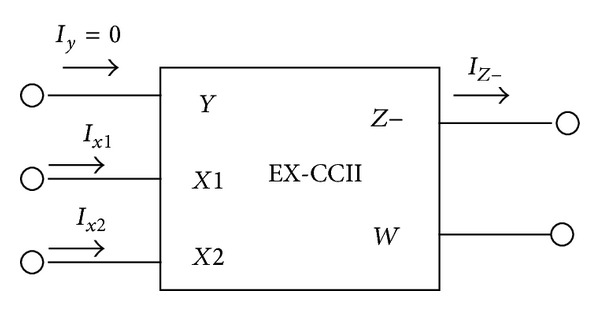
Symbol of EXCCII with buffered output.

The RC network in both [1] and (Filter-4 of [16]) is the same, but the active element is quite different. For this particular passive network, DXCCII is not a necessity, as simpler alternatives are possible. The basic scheme used in [1] is shown in Figure 1, which requires three voltage followers (VFs) and one current follower (CF) if all the features of [16] are also to be retained. Alternatively, a simpler circuit would require two voltage followers (one of them with two outputs) and one current follower. Now, lot of circuit complexity of “DXCCII with buffered output” used in [16] becomes redundant for realizing the goal. A simpler approach would only demand an extra-*X*  stage, resulting in a new active element, named EX-CCII (with buffered output), as DX is already reserved for dual-X CCII. As compared to a DXCCII, an EX-CCII has both  *X*1 and  *X*2 terminal voltage of same polarity as that of terminal  *Y*. The symbol of the newly introduced active building block is shown in Figure 2, with its defining equation as

(1) $$\begin{array}{c} I_{Y}=0,\;\;V_{X1}=V_{X2}=V_{Y},\\ I_{Z-}=-I_{X1},\;\;V_{W}=V_{Z}. \end{array}$$

**Figure 3:**
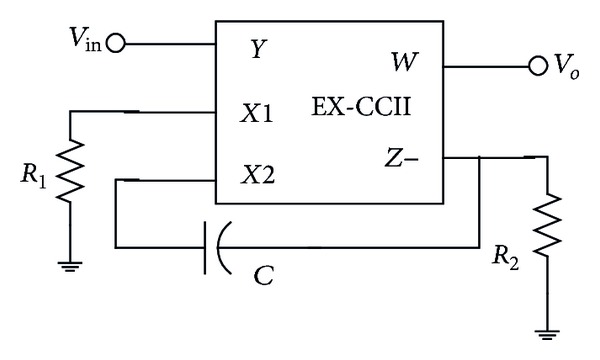
New proposed all-pass filter circuit.

With a view of the preceding developments, an optimum all-pass filter solution is now proposed in Figure 3. The voltage transfer function for the circuit is

(2) $$\begin{array}{c} T\left(s\right)=\frac{s-1/R_{1}C}{s+1/R_{2}C}. \end{array}$$

Using matched resistors (value  *R*), (2) above reduces to

(3) $$\begin{array}{c} T\left(s\right)=\frac{s-1/RC}{s+1/RC}. \end{array}$$

The phase function for the transfer function of (3) is

(4) $$\begin{array}{c} \Phi =180^{\circ }-2\text{ta}{\text{n}}^{-1}\left(\omega RC\right). \end{array}$$

**Figure 4:**
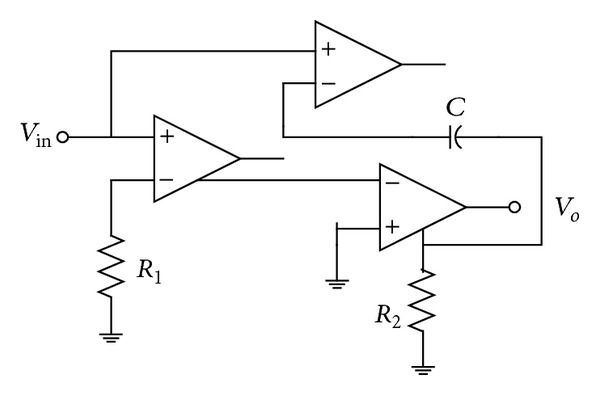
Realization of Figure 3 using AD-844s.

The circuit of Figure 3 considering [1, 16] is better than the latest one (Filter 4 of [16]) if circuit complexity is compared. Unlike DXCCII with buffered output, the new EXCCII-based circuit does not require  *X*_*n*_  stage. As far as the output buffer stage is concerned, it is the same as in [16], thus, making the new solution (Figure 3) complex in comparison to [1] but with additional advantages over [1] as already mentioned. This is what makes the proposal an optimum one keeping in view the two most time separated work [1, 16] by the author. Based on the realization of the circuit using VFs and CFs, the one in [1] requires a VF and a CF each. Similarly, the Filter-4 in [16] uses two VFs, two CFs, and an inverting-VF (IVF). It is to be noted that the new proposal is still an unreported circuit in the available literature on the topic [1–33]. It is also worth mentioning that the commercial realization of the filter 4 of [16] requires four AD-844 ICs as compared to three for the newly proposed solution, while retaining all the features of [16]. Recently, it was shown that a DXCCII-based circuit can be realized using four AD-844 ICs [34]. The three AD-844-based realization of the new circuit are shown in Figure 4. Therefore, the new solution is more compact than the latest one [16]. It may further be noted that the circuit of [1] can be realized using two AD-844 (it is well known that a CCII− requires two AD-844s for realization). Moreover, if the appropriate input and output features are to be imparted to the circuit in [1], then two additional AD-844 ICs would be required, each at the input and output end, making the count “four” but matching in other features with the proposed circuit in this work. Such a modified circuit may be referred to as (modified [1]), in the tabulated comparisons given in Table 1.

**Figure 5:**
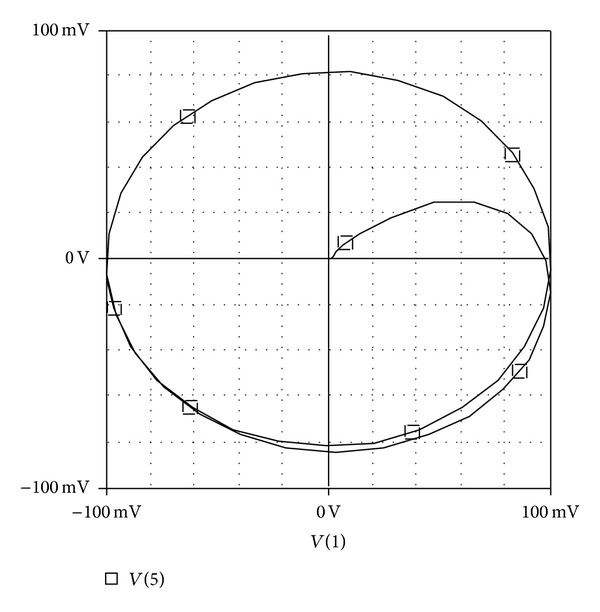
*X*-*Y*  plot showing 90° separations of input and output at pole frequency.

**Figure 6:**
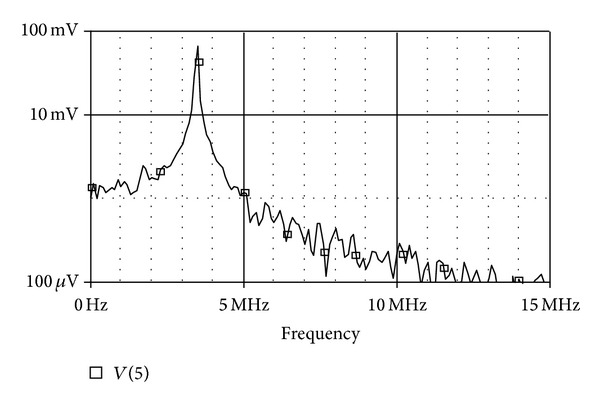
Output signal spectrum at pole frequency.

Although the inclusion of results was not the aim of this paper, but for sake of completeness, the new proposed EX-CCII is implemented using commercially available IC: AD-844 model. The implementation of the circuit of Figure 3 using AD-844 model is now employed for presentation of results. For simulations, the standard circuit simulator PSPICE is used. It may be clarified that the implementation of current conveyor with extra  *X*-stage is not yet available in literature. The circuit was configured with 10 K*Ω*  resistors and 5 pF capacitor so as the yield an input-output (*X*-*Y*) plot of the circuit at pole frequency of 3.3 MHz, as shown in Figure 5, which shows 90 degrees phase shift by virtue of being circular. Use of all-pass filter as phase shifter in communications is known since long time back [20]. Next, the Fourier spectrum of the output signal is also given in Figure 6, showing high selectivity of the signal for the pole-frequency.

## 4. Conclusive Discussion 

The motivation behind this work was to survey the current conveyor-based notable first-order all-pass sections proposed during the last decade. In the process, an optimum solution for this electronic function emerged, which proved to be another novel addition based on a newly coined active element, EXCCII. The work surveyed [1–16] are only a part of an otherwise huge sea of knowledge available on this electronic function. A brief listing of some of the other notable is very much in order, so as to complete this study. It may be noted that huge number of contributions are available, out of which the ones listed herein [17–33, 35–39] are significant, though it does not imply any insignificance to an equal number or more of the ones not cited. The new EXCCII-based circuit is found to possess advantages over the existing works. Moreover, the new active element imparts more versatility to the first invented CCII [40] and is expected to find more analog signal processing applications. As far as this work is concerned, it does serve the purpose of being an initiative for broader future study on the topic, besides coining a novel solution and simultaneously providing an insight to the topic for upcoming researchers in the area for coming decades.

## Figures and Tables

**Table 1:** Comparative study.

Circuit	VFs required	IVFs required	CFs required	Appropriate input, output impedances	AD-844s required for breadboarding
[1]	1	0	1	No	2
Modified [1]	3	0	1	Yes	4
Filter-4 in [16]	2	1	2	Yes	4
Proposed	3	0	1	Yes	3
